# Tocilizumab and rituximab for systemic sclerosis interstitial lung disease: a real-world cohort analysis

**DOI:** 10.1093/rheumatology/keaf006

**Published:** 2025-01-03

**Authors:** Nina R Goldman, Svetlana I Nihtyanova, Claire F Beesley, Athol U Wells, Christopher P Denton, Elisabetta A Renzoni, Rizgar Mageed, Voon H Ong

**Affiliations:** Division of Medicine, Centre for Rheumatology, University College London, London, UK; Division of Medicine, Centre for Rheumatology, University College London, London, UK; Clinical Research, Immunology, GSK, London, UK; Division of Medicine, Centre for Rheumatology, University College London, London, UK; Interstitial Lung Disease Unit, Royal Brompton Hospital, Guy’s and St Thomas’ Hospital NHS Foundation Trust, London, UK; Division of Medicine, Centre for Rheumatology, University College London, London, UK; Interstitial Lung Disease Unit, Royal Brompton Hospital, Guy’s and St Thomas’ Hospital NHS Foundation Trust, London, UK; Margaret Turner-Warwick Centre for Fibrosing Lung Disease, Imperial College London, London, UK; Centre for Translation Medicine and Therapeutics, Queen Mary University, London, UK; Division of Medicine, Centre for Rheumatology, University College London, London, UK

**Keywords:** anti-topoisomerase antibody, interstitial lung disease, rituximab, systemic sclerosis, tocilizumab

## Abstract

**Objectives:**

SSc-interstitial lung disease (ILD) is one of the leading causes of mortality in SSc. Data from randomized controlled trials (RCTs) support rituximab and tocilizumab monotherapy but there are limited data regarding their use for those who fail standard immunomodulatory therapies.

**Methods:**

SSc patients treated with rituximab or tocilizumab were retrospectively identified in a single centre cohort. Linear mixed effect models were used to analyse before and after treatment lung function trajectory, and identify patient characteristics associated with treatment response.

**Results:**

A total of 127 patients were included for analysis. Fifty-one of 94 (54.2%) and 13 of 33 (39.4%) of the rituximab and tocilizumab cohorts, respectively, were receiving concurrent MMF. Pre-treatment decline in absolute change % forced vital capacity (%FVC)/year and % diffusion capacity for carbon monoxide (%DLCO)/year, respectively, was similar in both cohorts (−3.2% and −4.0% rituximab, and −3.2% and −3.6% tocilizumab). Both treatments resulted in lung function stabilization (%FVC/year and %DLCO/year: 1.2% and +0.2% rituximab cohort, 1.0% and 1.0% tocilizumab cohort). Anti-topoisomerase antibody (ATA)-positive patients had a significant response on %FVC/year to tocilizumab compared with ATA-negative patients. Gender had a significant impact on %FVC/year response to rituximab, with males responding to a greater degree than females. Age, ILD extent and skin subset had no impact on treatment response.

**Conclusion:**

Combination rituximab or tocilizumab with background immunosuppressive therapy is associated with stabilization in lung function trajectory among those who remain refractory to standard immunosuppressives. Specific patient characteristics have an impact on lung function response. Improved FVC response among ATA patients receiving tocilizumab validate data from RCTs.

Rheumatology key messages

- These real world data demonstrate improvement in %FVC trajectory following treatment with rituximab and tocilizumab in SSc interstitial lung disease.
- Specific patients characteristics including anti-topoisomerase I antibody positivity and gender impact response to treatment.
- This data complements recent randomized controlled trials and supports the use of biologics in a more diverse patient cohort with severe SSc interstitial lung disease.

## Introduction

Interstitial lung disease (ILD) is a leading cause of mortality in SSc [1]. Male gender, older age, African American race, diffuse skin disease and anti-topoisomerase I antibody (ATA) positivity are reported risk factors for ILD development and progression [2] Low baseline forced vital capacity (FVC) and diffusion capacity for carbon monoxide (DLCO), CT disease extent over 20% and FVC decline of at least 10% are associated with progressive ILD [2]. However, the majority of studies do not consider predictors of progressive ILD treatment response.

Evidence from clinical studies support immunological approaches in SSc-ILD. The Scleroderma Lung Studies (SLS) indicate that CYC and MMF reduce ILD progression [3, 4]. Autologous stem cell transplant also provides supportive evidence [5]. Although there were initially mixed results, randomized controlled trials (RCTs) demonstrated improvement in FVC with rituximab, a chimeric mAb against human CD20 [6, 7]. A study with smaller SSc-ILD numbers showed short-term benefit with combination rituximab and MMF compared with MMF alone [8]. Monotherapy with tocilizumab, a mAb targeting the IL-6 receptor, was beneficial in arresting lung function decline in early-stage diffuse SSc [9, 10]. Post-hoc analysis of the phase III study confirmed early disease duration (<2 years), ATA positivity and male gender were predictive of tocilizumab response [11].

Real-world evidence can complement RCT evidence in treatment algorithms development and help in determining whether treatment benefits found in RCTs may extend to a broader SSc cohort. We therefore performed a retrospective cohort study to assess trajectory of ILD following treatment with rituximab or tocilizumab in SSc patients from our single-centre cohort, assessing differential response in subgroups based on patient characteristics.

## Methods

### Cohort selection

Patients from the Royal Free Hospital (London, UK) Scleroderma Cohort (SMART) who fulfilled the ACR/EULAR 2013 diagnostic criteria for SSc and had received one or more dose of rituximab (1000 mg at weeks 0 and 2) and/or ≥3 months tocilizumab with at least one pulmonary function test within 24 months before and after treatment were included [12]. Relevant demographic and clinical data were extracted from records.

The study was approved by London-Fulham NHS Research Ethics Committee (IRAS ID 279682) and all patients have provided written informed consent. This study complied with the ethical standards of the Declaration of Helsinki.

### Demographics, disease characteristics and outcomes

Disease onset was defined as time of first non-Raynaud’s manifestation of SSc, and early disease as ≤60 months since disease onset. Skin disease subtype by presence of skin involvement proximal or distal to the elbow or knees. Overlap syndromes were recorded. ILD was confirmed on high-resolution CT. Pulmonary hypertension, cardiac scleroderma, gastrointestinal involvement and scleroderma renal crisis were defined from previous SMART cohort studies [13]. Auto-antibody data were collected. If patients were positive for more than one antibody they were included in the antibody group specific to SSc.

All available pulmonary function tests were collected and included in analysis. The closest CRP prior to but within 12 months of starting biologic therapy was recorded. Elevated CRP was defined as ≥5 mg/l. Available CT scans of the chest performed within 12 months prior to starting treatment were scored using the Goh *et al.* extent of disease staging system by two independent assessors (N.R.G. and V.H.O.) [14]. Extensive disease was defined as ≥20% involvement.

If rituximab and tocilizumab had been given to the same patient, data was analysed for the first biologic only. Response to therapy was considered stabilization or improvement of lung function.

### Statistical analysis

Linear mixed effects models were used to describe the changes in absolute %FVC and %DLCO over 24 months pre- and post-treatment start. All available patient lung function within 24 months pre- and post-biological treatment were included. Patient characteristics and their interactions with time were included in the models as covariates to assess their effect on absolute %FVC and %DLCO change over time. For each effect we present ß coefficients, *P*-values and 95% CIs. *P*-value of ≤0.05 was deemed significant. Age was assessed as a continuous variable centred at 50 years. Statistical analysis was performed using Stata14.

## Results

### Description of study cohort

We identified 127 SMART cohort patients who received rituximab and/or tocilizumab. Eighty-seven had been treated with rituximab only, 32 tocilizumab only, and 8 received both biologics (7 rituximab initially and 1 tocilizumab initially). Mean number of available lung function per patient over the study period was 5.16 (range 2–13) and 4.45 (range 2–8) for the rituximab and tocilizumab group, respectively. Patients commenced rituximab between 2008 and 2021 and tocilizumab between 2013 and 2021.

Some 43.6% (*n* = 41) and 45.5% (*n* = 15) of the rituximab and tocilizumab patients, respectively, were ATA positive. At the time of biologic treatment, patients were relatively evenly split between early and late disease in both treatment groups [early disease: 45.7% (*n* = 43) rituximab, 57.6% (*n* = 19) tocilizumab]. The majority of patients received concurrent immunosuppression with their biologic therapy [rituximab: DMARD(s) 74.5% (*n* = 70), prednisolone 74.5% (*n* = 70), neither 5.3% (*n* = 5); tocilizumab: DMARD(s) 60.6% (*n* = 20), prednisolone 36.4% (*n* = 12), neither 27.3% (*n* = 9)], with MMF the predominant DMARD used. Prior CYC had been given to 43.6% (*n* = 41) of the rituximab and 18.2% (*n* = 6) of the tocilizumab cohort. One patient receiving tocilizumab had undergone autologous stem cell transplant for SSc. Of CT scans available (53 rituximab and 20 tocilizumab cohort), 26 (49.1%) of the rituximab and 3 (15%) of the tocilizumab patients had extensive disease pre-treatment. Additional cohort baseline characteristics are shown in Supplementary Table S1, available at *Rheumatology* online.

### Biologics impact on lung function trajectory

**Figure 1.**
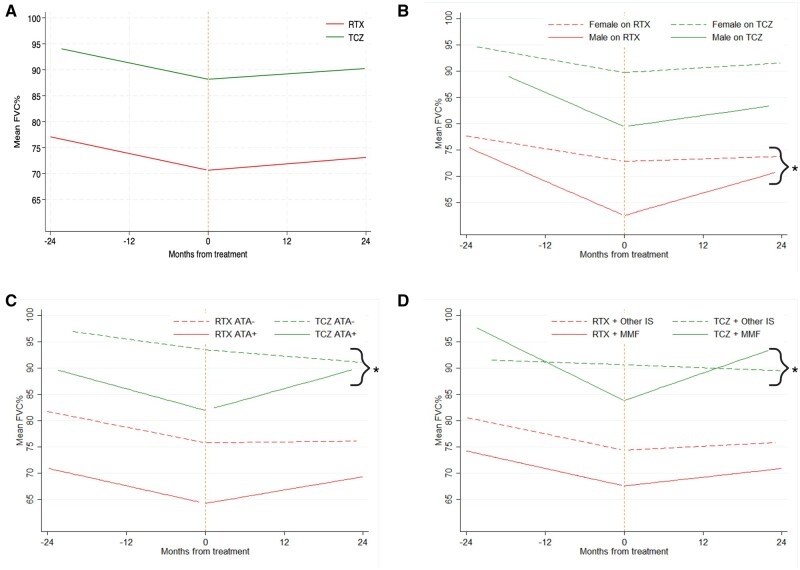
Change in %FVC over time pre and post treatment with rituximab or tocilizumab and interaction of other patient characteristics. Time 0 = time of biologic treatment. ^a^Significant effect of characteristics on annual rate of change in %FVC pre or post treatment compared with reference group (*P*-value ≤0.05). (**A**) Impact of treatment rituximab (RTX) or tocilizumab (TCZ). (**B**) Male or Female. (**C**) ATA+ *vs* ATA–. (**D**) concurrent MMF at treatment initiation *vs* other/no concurrent immunosuppression at treatment initiation (IS). Linear mixed model data is provided in Supplementary Tables S3 and S5, available at *Rheumatology* online. FVC: forced vital capacity; ATA: anti-topoisomerase antibody

Model-estimated mean %FVC and %DLCO at the time of treatment were lower in the rituximab-treated group compared with the tocilizumab-treated group (rituximab: %FVC 70.7, %DLCO 41.4; tocilizumab: %FVC 88.2%, %DLCO 60.5%) (Supplementary Table S2, available at *Rheumatology* online). Pre-treatment lung function decline was similar for both cohorts with change in %FVC/year and %DLCO/year in the rituximab cohort of –3.2% and –4%, respectively, and in the tocilizumab cohort of –3.2% and –3.6%, respectively (Fig. 1A, and Supplementary Table S3, available at *Rheumatology* online). Both treatments were associated with lung function stabilization with post-treatment change in %FVC/year and %DLCO/year, respectively, +1.2% and +0.2% for the rituximab cohort and +1.0% and +1.0% for the tocilizumab cohort (Supplementary Table S3, available at *Rheumatology* online).

### Impact of clinical characteristics on treatment response

Compared with females, males had a numerically greater pre-treatment decline in %FVC/year and lower %FVC on treatment initiation (Table 1, and Supplementary Table S2, available at *Rheumatology* online, and Fig. 1B). However, males demonstrated a greater improvement in %FVC with either biologic compared with females and responded better to rituximab compared with tocilizumab (Table 1).

**Table 1.** Effect of different patient characteristics on the yearly changes in %FVC in the two treatment cohorts pre- and post-treatment with rituximab or tocilizumab

Fixed effect parameter	Rituximab						Tocilizumab					
	Pre-treatment			Post-treatment			Pre-treatment			Post-treatment		
	Effect of characteristic on annual rate of change in %FVC^a^	*P*-value	95% CI	Effect of characteristic on annual rate of change in %FVC^a^	*P*-value	95% CI	Effect of characteristic on annual rate of change in %FVC^a^	*P*-value	95% CI	Effect of characteristic on annual rate of change in %FVC^a^	*P*-value	95% CI
Male	−4.14	0.08	−8.72, 0.44	**3.81**	**0.03**	**0.36, 7.27**	−3.92	0.46	−14.2, 6.37	1.23	0.74	−5.93, 8.38
dcSSc subset	−2.75	0.14	−6.36, 0.86	−0.03	0.98	−2.95, 2.89	−**10.6**	**0.04**	−**20.5,** −**0.69**	5.86	0.14	−1.91, 13.64
Inflammatory arthritis present	1.22	0.53	−2.63, 5.08	1.41	0.35	−1.52, 4.35	6.3	0.09	−0.98, 13.6	3.33	0.21	−1.83, 8.49
ATA positive	−0.37	0.85	−4.16, 3.42	2.37	0.11	−0.51, 5.26	−2.05	0.59	−9.40, 5.30	**5.34**	**0.05**	**0.02, 10.66**
Disease duration at treatment initiation ≤60 months	−2.04	0.27	−5.64, 1.57	−0.36	0.81	−3.25, 2.53	−1.75	0.64	−9.15, 5.65	−1.32	0.65	−7.05, 4.41
Concurrent MMF at treatment initiation	−0.22	0.91	−3.94, 3.49	0.91	0.53	−1.92, 3.75	−6.88	0.06	−14.1, 0.37	**5.79**	**0.04**	**0.33, 11.2**
Previous CYC	−1.82	0.34	−5.56, 1.92	1.65	0.25	−1.17, 4.4	−2.51	0.58	−11.4, 6.41	6.18	0.08	−0.08, 13.1
Pre-treatment CRP ≥5	−1.76	0.43	−6.13, 2.61	1.71	0.32	−1.62, 5.03	−7.2	0.10	−15.84, 1.45	0.07	0.98	−5.98, 6.11
Global extent on CT ≥20%	0.71	0.77	−4.11, 5.53	2.34	0.23	−1.44, 6.12	−3.49	0.58	−15.82, 8.84	10.45	0.07	−0.93, 21.82

Significant values are highlighted in bold.

a ß coefficient: effect of characteristic on annual rate of change in %FVC compared with reference group, e.g. male *vs* female, dcSSc *vs* lcSSc, inflammatory arthritis present *vs* absent, ATA positive *vs* negative. FVC: forced vital capacity; ATA: anti-topoisomerase antibody.

Diffuse compared with limited skin subset was associated with numerically lower absolute levels of %FVC and a significantly greater rate of decline pre-treatment in the tocilizumab cohort (Table 1, and Supplementary Table S2, available at *Rheumatology* online). However, no effect of skin subset on treatment response was observed (Table 1, and Supplementary Table S4, available at *Rheumatology* online). Similarly, age did not impact response to either therapy (data not shown).

Overlap inflammatory arthritis did not associate with %FVC or %DLCO values on treatment initiation or response to therapy. Disease duration did not appear to associate with either absolute %FVC levels or %FVC treatment response to rituximab or tocilizumab (Supplementary Fig. S1A, available at *Rheumatology* online). Early disease was associated with significantly faster decline in %DLCO pre-treatment in the rituximab group but not response to either treatment.

ATA-positive patients had significantly lower %FVC and %DLCO on treatment initiation compared with ATA-negative patients (Supplementary Table S2, available at *Rheumatology* online). While pre-treatment %FVC decline did not associate significantly with ATA positivity, treatment response did, with significantly greater response to tocilizumab and numerically greater but not statistically significant response to rituximab in %FVC in ATA-positive patients treated with rituximab compared with ATA-negative patients (Fig. 1C). The significantly greater %FVC response to tocilizumab in ATA-positive compared with ATA-negative patients was maintained when age and gender were included in the mixed effects model (ß = 5.8, *P* = 0.03, 95% CI 0.45–11.09).

Patients who had received prior CYC had a significantly lower starting %FVC (Supplementary Table S2, available at *Rheumatology* online). A numerically greater response was seen in tocilizumab patients who had received CYC previously, however this was a small patient subset (Table 1). Concurrent MMF use on biologic initiation was significantly associated with increased %FVC response to tocilizumab, but not rituximab (Fig. 1D). CRP level had no significant effect on response to either biologic (Supplementary Fig. S1B, available at *Rheumatology* online).

As expected, patients with global extent of disease >20% on CT had a lower %FVC on treatment initiation (Supplementary Table S2, available at *Rheumatology* online), although treatment response did not differ from those with less extensive disease.

## Discussion

In this analysis, rituximab and tocilizumab are associated with significant improvement in %FVC trajectory, including in those who remain refractory to standard immunosuppression extending data from recent RCTs [7, 9, 10]. ATA positivity and male gender significantly impacted %FVC treatment response to tocilizumab and rituximab, respectively. Two recent studies of real-world cohorts of tocilizumab in SSc reported a consistent but not significant effect on stabilization of %FVC; however, neither study identified predictors of treatment responsiveness [15, 16]. We showed that lung function stabilized in all patient subgroups independent of skin subset, disease duration, gender, inflammatory arthritis and inflammatory response.

In our cohort, baseline CRP was not predictive of response to tocilizumab and this is consistent with the focuSSced trial where high CRP level was prognostic for lung function decline but not predictive of treatment response [11]. A recent study reported immunosuppression stabilized lung function trajectory in patients with persistently elevated CRP, but worsened lung function decline in non-inflammatory patients. However, in this analysis, small numbers of patients were receiving MMF or rituximab and no patients were receiving tocilizumab [17].

Our data on differential response of ATA positive patients to tocilizumab is consistent with data from focuSSced post-hoc analysis. Of note, however, treatment response to both rituximab and tocilizumab occurred irrespective of ATA status in our cohort [11]. The differential response to rituximab in the ATA positive subgroup was not specifically addressed in the recent SSc-ILD rituximab monotherapy RCT [6].

Although we demonstrate a greater response among males only to rituximab, it should be noted that our tocilizumab analysis is underpowered. Post-hoc analyses from SLS I and II demonstrated men had a worse outcomes than women despite MMF or CYC [18]. The evaluation of efficacy and safety of rituximab with mycophenolate mofetil in patients with interstitial lung diseases (EVER-ILD) RCT did not report differential gender response with combination MMF and rituximab [8]. However, data from focuSSced showed that male gender predicted response to tocilizumab [11]. It is well established that male gender is associated with progressive SSc-ILD and along with ATA-positive patients rapid progressors may particularly benefit from additional biological therapies.

Few ILD therapeutic trials have evaluated outcomes with combination approaches. Upfront combination rituximab and MMF was associated with greater benefit on FVC change at 6 months compared with MMF alone in a cohort of patients with non-specific interstitial pneumonia, including a small number of SSc-ILD patient. Infection rates were higher with combination MMF and rituximab, however infections were predominantly non-serious viral infections [8]. The majority of our patients were on background DMARD therapy and we showed combination MMF with either rituximab or tocilizumab is beneficial. Notably the FVC response to tocilizumab–MMF was significant compared with tocilizumab treatment alone. The addition of tocilizumab may potentiate the effect of MMF and the timely introduction of a second agent may be particularly relevant in high-risk patients, however we are not aware of any published data regarding infection risk with combination therapy in SSc and this needs further research.

A high proportion, particularly of the rituximab-treated patients, had received prior CYC. Despite previous CYC, patients responded to treatment with rituximab and tocilizumab suggesting additive benefit of biologic treatment in patients with continued decline. Due to their recent approval, no patients were receiving anti-fibrotics.

The lack of effect of CT disease extent with biologic may relate to the small numbers. However, data on impact of disease extent on treatment response in SSc-ILD has not been consistent. Post-hoc analysis of SLS I suggested patients with more severe reticular changes on high-resolution CT may have a greater response to CYC [19]. However, the post-hoc focuSSced study analysis found no influence of quantitative ILD and fibrosis scores on baseline CT in tocilizumab response [20].

There are a number of study limitations. This is a single-centre retrospective data set, however this reflects a large well-characterized SSc cohort. Lung function testing frequency was variable and subject to clinical judgment, some characteristics were under-represented and there were a number of missing data points. It was not possible to clarify rituximab repeat doses, therefore the initial dose of rituximab was taken as rituximab start time. We were unable to reliably collect data on treatment complications.

Real-world treatment choice takes into account extrapulmonary conditions and patient comorbidities, and includes a more diverse patient cohort including patients with early and late disease, both skin subsets and of diverse ethnic backgrounds. Our data suggest stabilization of lung function decline with both rituximab and tocilizumab in a real-world SSc cohort. We propose tocilizumab in combination with MMF may be of particular benefit in patients who are ATA positive. However, both biologic therapies appear to stablize lung function decline in patients with both early and late disease who may have failed initial therapy.

## Supplementary Material

keaf006_Supplementary_Data

## Data Availability

The data underlying this article will be shared on reasonable request to the corresponding author.
