# Supplementary material for: Tocilizumab and rituximab for systemic sclerosis interstitial lung disease: a real-world cohort analysis
Source: Rheumatology (Oxford). 2025 Jan 3;64(SI):SI184–9. doi: 10.1093/rheumatology/keaf006 (PMC12695047; doi:10.1093/rheumatology/keaf006)
Supplement: keaf006_Supplementary_Data [file keaf006_supplementary_data.zip › 840e8_rhe-24-1360-File003.docx]

**Supplementary material**

**Supplementary Table S1:** Baseline demographic and clinical characteristics of the cohort

|  | Rituximab | Tocilizumab | Total |
| --- | --- | --- | --- |
| **Patients, n (%)** | 94 (70.7) | 33 (24.8) | 127 |
| **Gender** Female, N (%) | 74 (78.7) | 28 (84.8) | 102 (80.3) |
| **Ethnicity*** Caucasian, N (%) | 47 (50) | 21 (63.6) | 68 (53.5) |
| Black, N (%) | 17 (18.1) | 3 (9.09) | 20 (15.8) |
| Asian, N (%) | 17 (18.1) | 5 (15.2) | 22 (17.3) |
| Mixed / Other, N (%) | 7 (7.5) | 3 (9.09) | 10 (7.9) |
| **Smoking history** Ever smoker, N (%) | 20 (21.3) | 13 (39.4) | 33 (26) |
| **Cutaneous Subset:** Diffuse, N (%) | 48 (51.1) | 27 (81.8) | 75 (59.1) |
| **Overlap rheumatic features** Inflammatory arthritis, N (%) | 38 (40.4) | 20 (60.6) | 58 (45.7) |
| Myositis, N (%) | 36 (38.3) | 5 (15.2) | 41 (32.3) |
| Vasculitis, N (%) | 7 (7.5) | 0 | 7 (5.5) |
| Systemic Lupus Erythematosus, N (%) | 6 (6.4) | 0 | 6 (4.7) |
| MCTD, N (%) | 1 (1.1) | 0 | 1 (0.8) |
| Sjogren’s syndrome, N (%) | 7 (7.5) | 0 | 7 (5.5) |
| **Autoimmune serology^±^** ANA, N (%) | 93 (98.9) | 33 (100) | 126 (99.2) |
| Anti-centromere, N (%) | 6 (6.4) | 1 (3.0) | 7 (5.5) |
| Anti-topoisomerase, N (%) | 41 (43.6) | 15 (45.5) | 56 (44.1) |
| Anti-RNA polymerase, N (%) | 3 (3.2) | 10 (3.0) | 13 (10.2) |
| ANA + ENA -, N (%) | 9 (9.6) | 3 (9.1) | 12 (9.4) |
| Other^§^, N (%) | 30 (31.9) | 3 (9.1) | 33 (26) |
| **Organ involvement** ILD, N (%) | 81 (86.2) | 21 (63.6) | 102 (80.3) |
| PH (Group 1 & Group 3), N (%)  *Group 1 PAH*, N (%) | 16 (17)  10 (62.5) | 2 (6.1)  2 (100) | 18 (14.2)  12 (66.7) |
| Cardiac SSc, N (%) | 12 (12.8) | 0 | 12 (9.5) |
| Scleroderma Renal crisis, N (%) | 0 | 1 (3.03) | 1 (0.79) |
| Gastrointestinal involvement, N (%) | 51 (54.3) | 11 (33.3) | 62 (48.8) |
| **Treatment timing^#^** Median age at disease onset (years), (range) | 42 (16 – 67) | 43.5 (6 – 67) | 42 (6-67) |
| Median age at therapy start (years), (range) | 50 (19 – 80) | 51 (16 – 69) | 50 (16 – 80) |
| Months from disease onset to therapy start, Median (range) | 73 (9 – 390) | 29.5 (11 – 201) | 62 (9 – 390) |
| Time from disease onset to therapy start, ≤60 months, N (%) | 43 (45.7) | 19 (57.6) | 62(48.8) |
| **Medication at treatment initiation** Mycophenolate Mofetil, N (%) | 51 (54.3) | 13 (39.4) | 64 (50.4) |
| Prednisolone , N (%) | 70 (74.5) | 12 (36.4) | 82 (64.6) |
| Hydroxychloroquine, N (%) | 29 (30.9) | 8 (24.2) | 37 (29.1) |
| Other^$^, N (%) | 17 (18.1) | 6 (18.2) | 24 (18.9) |
| No concurrent immunosuppression, N (%) | 5 (5.3) | 9 (27.3) | 14 (11) |
| Nintedanib or pirfenidone, N (%) | 0 | 0 | 0 |
| **Pre-Treatment blood results^†^** CRP≥5, N (%) | 37 (39.4) | 12 (36.4) | 49 (38.6) |
| **Died within 24 months of therapy start N (%)** | 2 (2.1) | 0 | 2 (1.6) |
| *. Missing ethnicity data for 6 patients from the rituximab group and 1 from the tocilizumab group  **±**. Missing data for patients for antibodies for 1 tocilizumab patient. For 3 rituximab patients ANA+/ENA+ but no specific ENA data available.  §. Other includes anti PmScl, Th/To, Ro-52, nRNP, u3RNP, Ro, La, PL7, Ku, Sm, XR  #. Missing data disease onset for 1 patient from the rituximab group and 1 from the tocilizumab group  $. Includes methotrexate, azathioprine, ciclosporin  †. Missing blood result data for 27 patients from the rituximab and 8 from the tocilizumab group | | | |

**Supplementary Table S2:** Model estimated mean lung function at treatment initiation^*^:

|  | | Rituximab | | Tocilizumab | |
| --- | --- | --- | --- | --- | --- |
|  |  | %FVC | %DLCO | %FVC | %DLCO |
| All patients | | 70.7 | 41.4 | 88.2 | 60.5 |
| Gender | Male | **62.5** | 39.6 | **79.4** | 58.7 |
|  | Female | 72.8 | 41.9 | 89.7 | 61 |
| Cutaneous subset | dcSSc | 69 | 43.7 | 88 | 61.3 |
|  | lcSSc | 72.2 | 39 | 91.2 | 56.7 |
| Inflammatory arthritis | Yes | 71.2 | 43.6 | 88.4 | 62.6 |
|  | No | 70.2 | 39.9 | 87.4 | 59 |
| ATA | ATA+ | **64.3** | **37.1** | **81.9** | **57.1** |
|  | ATA- | 75.8 | 44.6 | 93.4 | 64.6 |
| Disease duration at treatment initiation | ≤60 months | 70.2 | 43.3 | 86.1 | 62.9 |
|  | >60 months | 70.6 | 39.1 | 86.5 | 58.6 |
| MMF at treatment initiation | MMF | 67.6 | **38.2** | 83.8 | **56.4** |
|  | No MMF | 74.4 | 45.2 | 90.6 | 63.4 |
| Previous cyclophosphamide | Yes | **65.8** | 38.6 | **81** | 56.3 |
|  | No | 74.7 | 43.9 | 89.9 | 61.5 |
| Pre-treatment CRP | >5 | 67.3 | 37.1 | 80.8 | 59.7 |
|  | ≤5 | 69.8 | 40.1 | 83.3 | 62.6 |
| Global extent on pre-treatment CT | >20% | **59.3** | **31.7** | **73.8** | **49.4** |
|  | ≤20% | 74.6 | 47.2 | 89.1 | 64.9 |
| *: Where differences are statistically significant between groups eg male vs females, values are highlighted with bold text | | | | | |

**Supplementary Table S3:** Effect of rituximab and tocilizumab treatment on annual rate of change in %FVC – results from linear mixed effect models.

|  | | ß | P value | 95% CI |
| --- | --- | --- | --- | --- |
| Pre-biologic | Rituximab | **-3.22** | **0.001** | **-5.09, -1.36** |
|  | Tocilizumab | -3.15 | 0.09 | -6.83, 0.52 |
| Post-biologic | Rituximab | 1.23 | 0.09 | -0.21, 2.67 |
|  | Tocilizumab | 1.03 | 0.42 | -1.49, 3.56 |
|  | | | | |
|  | Constant | **70.66** | **<0.001** | **66.48, 74.83** |
|  | Tocilizumab | **17.52** | **<0.001** | **9.21, 25.83** |

**Supplementary Table S4:** Effect of different patient characteristics on the changes in %DLCO over time in the two treatment cohorts pre and post treatment with rituximab or tocilizumab

| Fixed effect parameter | Rituximab | | | | | | Tocilizumab | | | | | |
| --- | --- | --- | --- | --- | --- | --- | --- | --- | --- | --- | --- | --- |
|  | Pre-Treatment | | | Post Treatment | | | Pre-Treatment | | | Post-Treatment | | |
|  | Effect of characteristic on annual rate of change in %DLCO^*^ | *P* value | 95% CI | Effect of characteristic on annual rate of change in %DLCO^*^ | *P* value | 95% CI | Effect of characteristic on annual rate of change in %DLCO^*^ | *P* value | 95% CI | Effect of characteristic on annual rate of change in %DLCO^*^ | *P* value | 95% CI |
| Male | -3.23 | 0.12 | -7.25, 0.8 | 0.68 | 0.72 | -3.03, 4.4 | 0.81 | 0.86 | -8.1, 9.72 | -3.63 | 0.33 | -10.94, 3.68 |
| dcSSc subset | **-3.56** | **0.03** | **-6.76, -0.36** | 1.39 | 0.37 | -1.63, 4.41 | -4.12 | 0.34 | -12.60, 4.37 | 0.92 | 0.83 | -7.61, 9.45 |
| Inflammatory Arthritis | 1.4 | 0.42 | -1.96, 4.76 | -0.76 | 0.63 | -3.9, 2.37 | 5.86 | 0.07 | -0.42, 12.14 | -2.25 | 0.41 | -7.6, 3.09 |
| ATA | -0.98 | 0.56 | -4.29, 2.33 | -0.97 | 0.54 | -4.02, 2.08 | 0.99 | 0.76 | -5.33, 7.32 | 0.61 | 0.82 | -4.67, 5.89 |
| Disease duration at treatment initiation ≤60m | **-4.32** | **0.01** | **-7.65, -0.99** | 1.23 | 0.44 | -1.90, 4.35 | 0.35 | 0.92 | -6.05, 6.75 | -1.66 | 0.58 | -7.55, 4.22 |
| Concurrent MMF at treatment initiation | -0.93 | 0.58 | -4.23, 2.38 | 0.15 | 0.93 | -2.92, 3.22 | -4.68 | 0.15 | -10.98, 1.61 | -1.13 | 0.7 | -6.9, 4.63 |
| Previous cyclophosphamide | -3.08 | 0.06 | -6.33, 0.18 | 2.27 | 0.14 | -0.74, 5.27 | -1.69 | 0.66 | -9.3, 5.92 | -0.36 | 0.93 | -8.14, 7.43 |
| Pre-treatment CRP ≥5 | 0 | 1 | -4.33, 4.33 | 0.34 | 0.82 | -2.62, 3.31 | 1.89 | 0.64 | -6.1, 9.89 | 0.16 | 0.95 | -5.25, 5.57 |
| Global extent on CT ≥20% | 0.44 | 0.83 | -3.59, 4.46 | 1.67 | 0.47 | -2.88, 6.21 | -2.42 | 0.64 | -12.4, 7.57 | 5.76 | 0.39 | -7.25, 18.76 |
| Significant values are highlighted in bold  * ß coefficient, Effect of characteristic on annual rate of change in %DLCO compared to reference group eg male vs female, dcSSc vs lcSSc, inflammatory arthritis present vs absent, ATA positive vs negative | | | | | | | | | | | | |

**Supplementary Table S5:** Impact of different clinical characteristics on annual rate of change in %FVC pre and post treatment with rituximab and tocilizumab – results from linear mixed effect models. a) male vs female gender, b) effect of ATA+ vs ATA- c) concurrent MMF at treatment initiation vs other/no concurrent immunosuppression at treatment initiation, d) Disease duration ≤60m or >60m, e) CRP ≥5 or <5

| **S5a:** | | **ß** | **P value** | **95% CI** |
| --- | --- | --- | --- | --- |
| Pre-biologic: Females | Rituximab | -2.42 | 0.02 | -4.48, -0.35 |
|  | Tocilizumab | -2.62 | 0.19 | -6.54, 1.31 |
| Post-biologic: Females | Rituximab | 0.47 | 0.56 | -1.10, 2.05 |
|  | Tocilizumab | 0.90 | 0.50 | -1.75, 3.56 |
| Pre-biologic: Males | Rituximab | -4.14 | 0.08 | -8.72, 0.44 |
|  | Tocilizumab | -3.92 | 0.46 | -14.22, 6.37 |
| Post-biologic: Males | Rituximab | **3.81** | **0.03** | **0.36, 7.27** |
|  | Tocilizumab | 1.23 | 0.74 | -5.93, 8.39 |
|  | | | | |
|  | Constant | **72.83** | **<0.001** | **68.31, 77.36** |
|  | Tocilizumab | **16.89** | **<0.001** | **8.70, 25.07** |
|  | Male | **-10.29** | **0.02** | **-19.20, -1.39** |

| **S5b** | | | | |
| --- | --- | --- | --- | --- |
| Pre-biologic: ATA- | Rituximab | -2.98 | 0.03 | -5.57, -0.38 |
|  | Tocilizumab | -2.06 | 0.45 | -7.45, 3.32 |
| Post-biologic: ATA- | Rituximab | 0.16 | 0.88 | -1.85, 2.17 |
|  | Tocilizumab | -1.20 | 0.47 | -4.42, 2.03 |
| Pre-biologic: ATA+ | Rituximab | -0.37 | 0.85 | -4.16, 3.42 |
|  | Tocilizumab | -2.05 | 0.59 | -9.40, 5.3 |
| Post-biologic: ATA+ | Rituximab | 2.37 | 0.11 | -0.51, 5.26 |
|  | Tocilizumab | **5.34** | **0.05** | **0.02, 10.66** |
|  | | | | |
|  | Constant | **75.78** | **<0.001** | **70.65, 80.91** |
|  | Tocilizumab | **17.67** | **<0.001** | **9.48, 25.85** |
|  | ATA+ | **-11.52** | **0.001** | **-18.60, -4.44** |

| **S5c** | | | | |
| --- | --- | --- | --- | --- |
| Pre-biologic: other/no concurrent immunosuppression at treatment initiation | Rituximab | **-3.11** | **0.03** | **-5.95, -0.27** |
|  | Tocilizumab | -0.52 | 0.83 | -5.32, 4.28 |
| Post-biologic: other/no concurrent immunosuppression at treatment initiation | Rituximab | 0.75 | 0.49 | -1.40, 2.90 |
|  | Tocilizumab | -0.57 | 0.70 | -3.47, 2.32 |
| Pre-biologic: MMF at treatment initiation | Rituximab | -0.22 | 0.91 | -3.94, 3.50 |
|  | Tocilizumab | -6.88 | 0.06 | -14.12, 0.37 |
| Post-biologic: MMF at treatment initiation | Rituximab | 0.91 | 0.53 | -1.92, 3.75 |
|  | Tocilizumab | **5.79** | **0.04** | **0.33, 11.25** |
|  | | | | |
|  | Constant | **74.37** | **<0.001** | **68.64, 80.10** |
|  | Tocilizumab | **16.24** | **<0.001** | **7.96, 24.53** |
|  | MMF at treatment initiation | -6.80 | 0.06 | -14.00, 0.40 |

| **S5d** | | | | |
| --- | --- | --- | --- | --- |
| Pre-biologic: Disease duration at treatment initiation >60m | Rituximab | **-2.69** | **0.02** | **-4.92, -0.45** |
|  | Tocilizumab | -3.72 | 0.22 | -9.67, 2.24 |
| Post-biologic: Disease duration at treatment initiation >60m | Rituximab | 1.50 | 0.12 | -0.38, 3.39 |
|  | Tocilizumab | 2.76 | 0.28 | -2.21, 7.73 |
| Pre-biologic: Disease duration at treatment initiation ≤60m | Rituximab | -2.04 | 0.27 | -5.64, 1.57 |
|  | Tocilizumab | -1.75 | 0.64 | -9.15, 5.65 |
| Post-biologic: Disease duration at treatment initiation ≤60m | Rituximab | -0.36 | 0.81 | -3.25, 2.53 |
|  | Tocilizumab | -1.32 | 0.65 | -7.05, 4.41 |
|  | | | | |
|  | Constant | **70.56** | **<0.001** | **65.28, 75.84** |
|  | Tocilizumab | **15.92** | **<0.001** | **7.52, 24.32** |
|  | Disease duration at treatment initiation ≤60m | -0.38 | 0.92 | -7.63, 6.87 |

| **S5e** | | | | |
| --- | --- | --- | --- | --- |
| Pre-biologic: CRP<5 | Rituximab | -2.59 | 0.12 | -5.82, 0.64 |
|  | Tocilizumab | -2.80 | 0.36 | -8.76, 3.15 |
| Post-biologic: CRP <5 | Rituximab | 0.21 | 0.86 | -2.15, 2.57 |
|  | Tocilizumab | 3.04 | 0.19 | -1.51, 7.58 |
| Pre-biologic: CRP ≥5 | Rituximab | -1.76 | 0.43 | -6.13, 2.61 |
|  | Tocilizumab | -7.20 | 0.10 | -15.84, 1.45 |
| Post-biologic: CRP ≥5 | Rituximab | 1.71 | 0.32 | -1.62, 5.03 |
|  | Tocilizumab | 0.07 | 0.98 | -5.98, 6.11 |
|  | | | | |
|  | Constant | **69.82** | **<0.001** | **63.36, 76.28** |
|  | Tocilizumab | **13.44** | **0.004** | **4.21, 22.67** |
|  | CRP>=5 | -2.48 | 0.55 | -10.57, 5.61 |

**Supplementary Data S1:** Supplementary statistical method

Cohort characteristics at baseline, defined as the time of first treatment with a biologic, were summarised using descriptive statistics. Serial %FVC and %DLCO measurements from the 24-months before and after treatment start were modelled using linear mixed effects models, with random effects for intercept and slope. In those models, serial %FVC or % DLCO respectively were included as outcome. Type of biologic (rituximab or tocilizumab), patient and disease characteristics (sex, cutaneous subset, overlap features, autoantibody specificity, disease duration at baseline, concurrent MMF use, previous cyclophosphamide use, pre-treatment CRP, global extent of ILD on CT), time and the interactions of patient/disease characteristics and time, were included as predictor variables, to assess their effect on change over time in %FVC and %DLCO. Where interactions were not significant, those were dropped from the models. All lung function results available for each patient within the 24 months pre and post biological treatment were included. Initially, we assessed the rates of change in %FVC and %DLCO before and after biologic treatment in subgroups by type of biologic. Following this, we tested the effect each patient/disease characteristic has on the rates of change in %FVC and %DLCO within each biologic group. For each effect, we report the ß coefficient, 95% CIs and p-values estimated from the mixed models. A p-value of ≤0.05 is considered significant. Age was assessed as a continuous variable centered at 50 years. Statistical analysis was performed using Stata14.

**Supplementary Figure S1:** Change in %FVC pre and post treatment with rituximab or tocilizumab and interaction of other patient characteristics. Time 0 = time of biologic treatment. a) Disease duration ≤60m or >60m, b) CRP ≥5 or <5. Linear mixed model data is provided in Table S5.

**
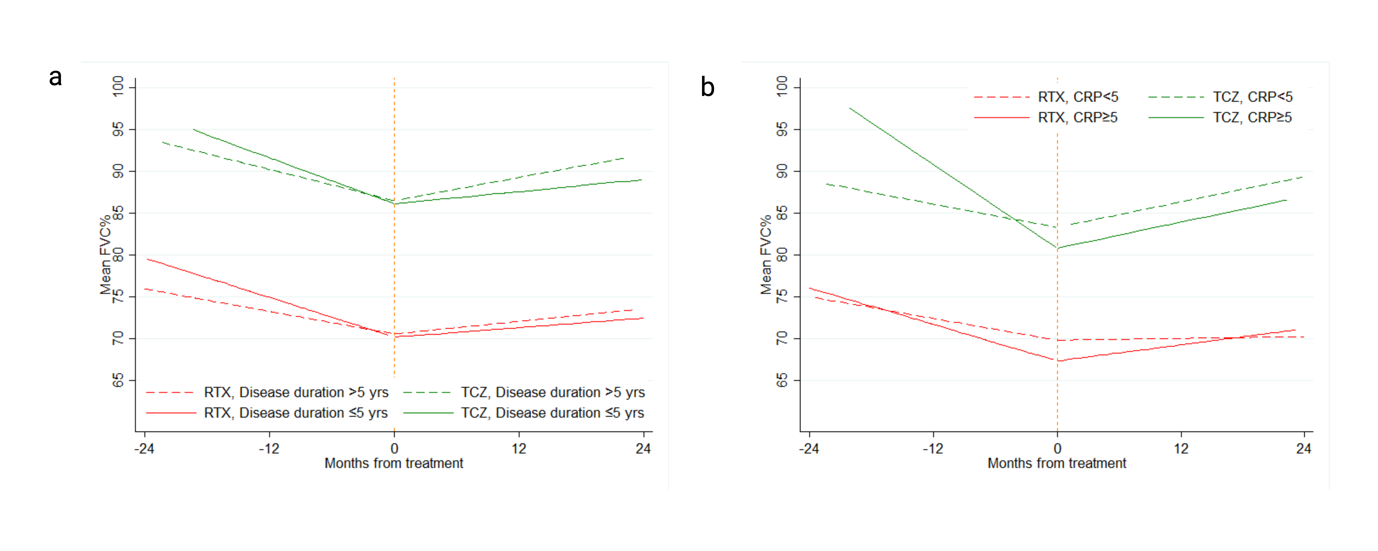
**
